# COVID-19 Infection and ST-Elevation Myocardial Infarction: Lessons in Disease Management During a Pandemic

**DOI:** 10.7759/cureus.62008

**Published:** 2024-06-09

**Authors:** Anna Carmichael, Joseph T Hale, Shahnawaz Notta, Tariq Haddadin, Manar H Jbara

**Affiliations:** 1 Cardiology, East Tennessee State University Quillen College of Medicine, Johnson City, USA; 2 Internal Medicine, East Tennessee State University Quillen College of Medicine, Johnson City, USA

**Keywords:** hemostasis and thrombosis, myocardial infarction, st-elevation myocardial infarction (stemi), sars-cov-2, covid-19

## Abstract

The emergence of the SARS-CoV-2 virus, causing the COVID-19 pandemic, has profoundly impacted global health, resulting in significant morbidity and mortality worldwide. This paper presents a case study highlighting the heightened risk of severe cardiovascular complications following COVID-19 infection. A 61-year-old male with hyperlipidemia was discharged after COVID-19 pneumonia treatment and experienced a severe ST-elevated myocardial infarction (STEMI) within a day of discharge. A retrospective chart review, supplemented by a literature review, revealed a pattern of increased severity in STEMI cases associated with COVID-19, particularly in patients with pre-existing cardiovascular comorbidities. SARS-CoV-2 induces a prothrombotic state, which causes endothelial dysfunction and systemic inflammation, potentially precipitating thrombotic events. Managing concurrent COVID-19 and STEMI poses unique challenges, emphasizing the critical role of timely intervention, such as percutaneous coronary intervention (PCI), in improving patient outcomes. Despite advancements, uncertainty persists regarding optimal thromboembolism prophylaxis post COVID-19, necessitating ongoing research and meticulous clinical management. While COVID-19 infection rates have declined since the pandemic, this case report hopes to emphasize the need for continued awareness in recognizing the potential thrombotic risks of COVID-19 infection and underscore the need for further investigation into cardiovascular risk as new viral strains develop in the future.

## Introduction

The SARS-CoV-2 virus, also known as the coronavirus disease of 2019 (COVID-19), has created a pandemic and led to over 6.9 million deaths globally since its discovery in 2019 [1]. While COVID-19 infection primarily causes acute respiratory distress, it has also been suggested to increase thrombus burden and worsen cardiovascular outcomes in concurrent ST-elevation myocardial infarction (STEMI) [2-4]. This case outlines the importance of close follow-up of patients with comorbidities and aims to increase awareness among clinicians of possible severe adverse cardiovascular outcomes after COVID-19 infection, as early detection is critical in preventing death. The following is a case report of a 61-year-old male with hyperlipidemia who suffered from a severe STEMI one day after discharge from hospitalization due to COVID-19 pneumonia.

## Case presentation

**Figure 1:**
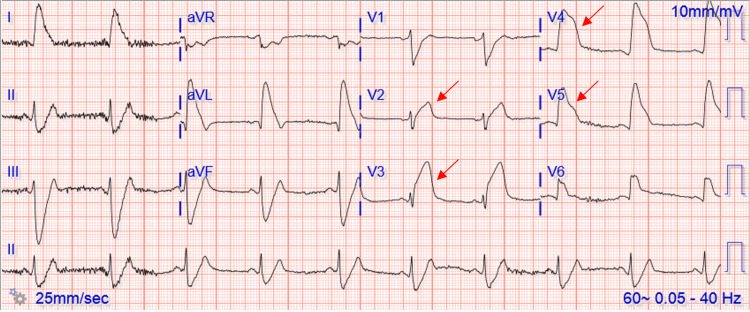
ST-elevation in the patient’s electrocardiogram

**Figure 2:**
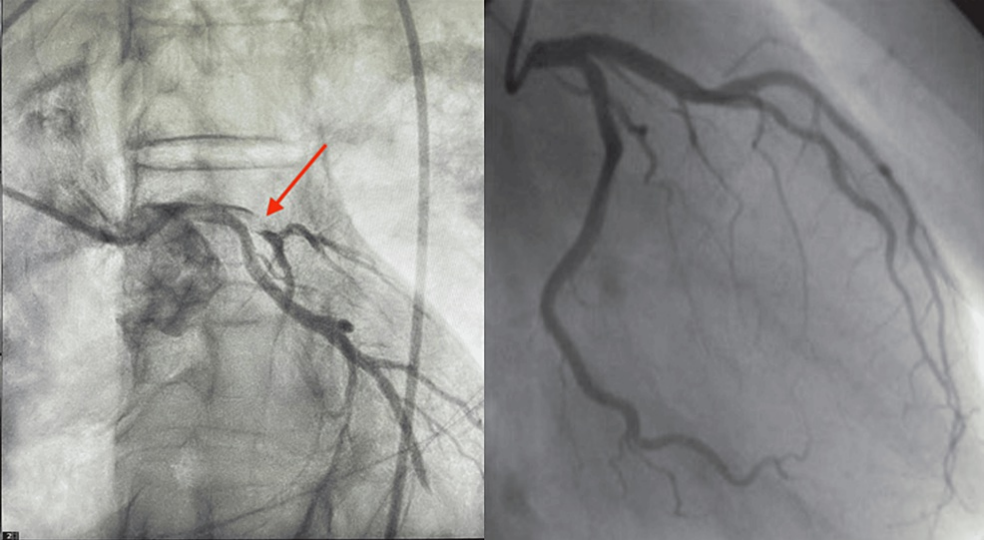
Patient’s coronary angiogram exhibiting thrombosis in the left coronary system (red arrow) (left) and non-pathologic filling of the left coronary system (right)

A 61-year-old white male was hospitalized with COVID-19 viral pneumonia. His past medical history included hyperlipidemia, and his family history was positive for stroke (mother) and heart disease (father). His social history was unremarkable. During hospitalization, the patient was treated with remdesivir, and after five days, the patient was discharged with supplemental oxygen. On the day following discharge, the patient urgently arrived at the emergency department by ambulance, expressing concerns about a sudden onset of chest pain characterized by sharp substernal discomfort that radiated to both arms. Accompanying symptoms included dyspnea, diaphoresis, and weakness. A STEMI was called in the field using an EKG (Figure 1), and the patient was given aspirin 324 mg before arrival and ticagrelor and heparin in the emergency department per the STEMI protocol. His heart rate was 55 beats/minute, respirations of 42 breaths/minute, and systolic blood pressure of 70mmHg. His TIMI score was 7, putting him at 23.4% risk of all-cause mortality at 30 days. The patient was taken immediately for catheterization where acute thrombosis in the left main coronary, left anterior descending, and circumflex arteries were found. Thrombectomy was performed on all occluded vessels, and a drug-eluting stent was placed in the left anterior descending artery by percutaneous coronary intervention (PCI), as seen in Figure 2.

During the procedure, the patient went into cardiac arrest with ventricular fibrillation requiring five rounds of defibrillation, after which a return of spontaneous circulation was achieved. The patient was placed on amiodarone, integrin, dopamine, and an intra-aortic balloon pump. On arrival at the intensive care unit, the patient’s ejection fraction was 30%, leading to cardiogenic shock, and the electrocardiogram noted ongoing ST elevations. His oxygen saturations were in the 80s, and he was placed on a non-rebreather mask. At this time, his prothrombin time (PT) was 13.7, and his activated partial PT (aPTT) was 23.7. Over the course of his intensive care unit stay, the patient was carefully hemodynamically monitored and began progressing well. He was transferred out of the intensive care unit to inpatient care after two days where he continued to improve. Following a comprehensive five-day hospitalization, the patient was released with a prescribed regimen comprising apixaban 5 mg BID, atorvastatin 80 mg QD, metoprolol succinate 12.5 mg QD, ticagrelor 90 mg BID, and aspirin 81 mg QD. Additionally, the patient received a life vest as part of the post-discharge care plan.

## Discussion

The current literature supports that STEMI in patients with COVID-19 is more severe than STEMI in patients without the infection, and urgent PCI continues to be the best treatment option [5]. Patients with concomitant STEMI and COVID-19 infection have been shown to have worse hospital outcomes compared to those not infected. Comorbidities that are associated with worse outcomes include older age, male sex, and comorbidities, including cardiovascular disease, hypertension, diabetes, chronic pulmonary disease, and cancer [6]. The prothrombotic state of patients with COVID-19 requiring hospitalization seems to play a large role in the worsening of cardiovascular outcomes [7]. However, the literature shows that many other factors are at play, resulting in increased adverse cardiovascular outcomes in patients with severe COVID-19, including fibrotic, increased reactive oxygen species, hypertrophic, vasoconstrictive, and gut dysbiosis effects caused by the immune system’s reaction to the virus [8]. Although our patient survived, he did suffer from a severe STEMI with multiple complications during his hospital stay. The importance of screening patients hospitalized for severe COVID-19 for cardiovascular complications remains crucial to prevent mortality in patients predisposed to these complications.

Pathophysiology

**Figure 3:**
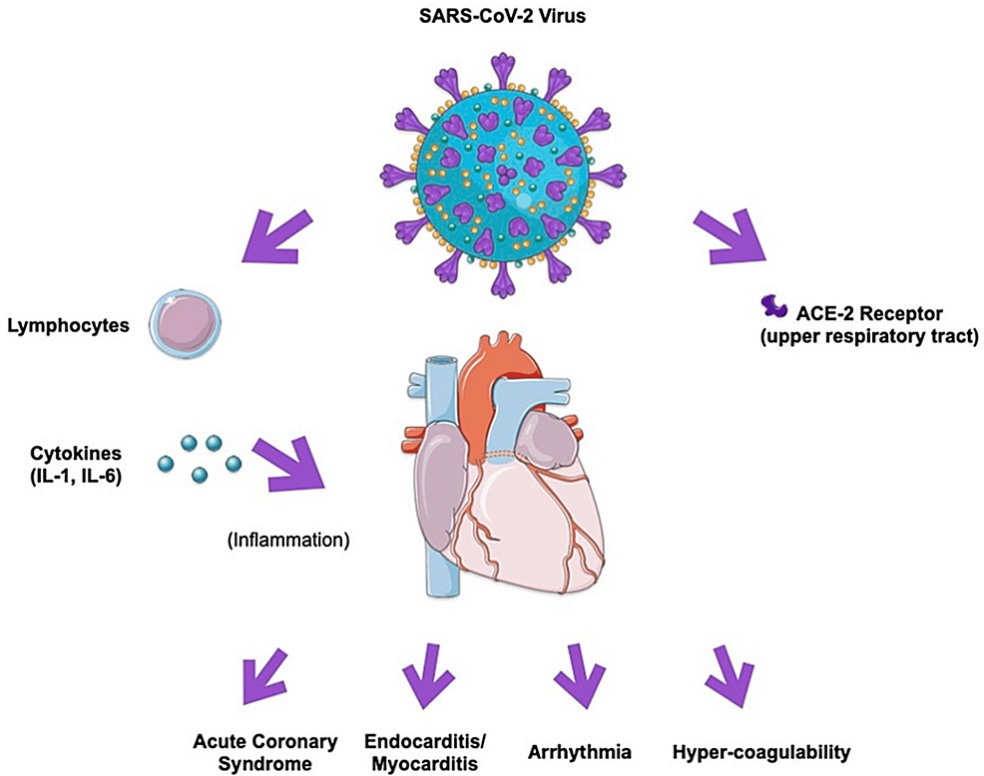
SARS-CoV-2 virus' pathophysiology Parts of the figure were drawn by using pictures from Servier Medical Art. Servier Medical Art by Servier is licensed under a Creative Commons Attribution 3.0 Unported License (https://creativecommons.org/licenses/by/3.0/ accessed on 7 July 2023).

During homeostasis, the vascular endothelium is rarely prothrombotic. However, infection with the SARS-CoV-2 virus has the potential to trigger the release of a multitude of inflammatory cytokines that turn the endothelium procoagulant, thereby increasing the risk of thromboembolic events, such as in our patient. The activated endothelium then releases tissue factor, thromboxane, and von Willebrand factor, which activate the clotting cascade and constrict blood vessels. Fibrinolysis is also antagonized by the release of plasminogen activator inhibitor (PAI)-1 [8]. SARS-CoV-2 infection enters cells through angiotensin-converting enzyme (ACE)-2 receptors, which are expressed in nasal and bronchial epithelial cells and type II alveolar pneumocytes [8]. The critical threshold between SARS-CoV-2 infection and severe SARS-CoV-2 infection is the involvement of the vascular endothelium. Although cytokine recruitment is mainly to areas of infection and surrounding cells (in this case, the lungs and airway passages), SARS-CoV-2 infection also causes systemic hypercoagulability activated by IL-1 and IL-6, which increases hepatocyte production of fibrinogen, PAI-1, and CRP, thereby increasing systemic inflammation and circulation of clotting and antifibrinolytic factors (Figure 3). Along with hypercoagulability, viral infection with SARS-CoV-2 and release of pathogen-associated patterns (PAMPs) activate molecular markers on atherosclerotic plaques that predispose them to rupture, increasing the risk for STEMI. The mentioned systemic effects can also reach the coronary vessels, increasing the risk for vasoconstriction and thrombosis at the heart specifically [8].

Disease management

Cardiovascular complications of SARS-CoV-2 infection can include pericarditis, myocarditis, and elevated troponin and brain natriuretic peptide (BNP) levels with varying degrees of myocardial injury. This can make the diagnosis of concomitant acute coronary syndrome difficult [9]. The most important factor in treating patients with concomitant SARS-CoV-2 infection and STEMI is to decrease the door-to-balloon time. Rapid access to PCI is the most effective way to decrease long-term complications and mortality in STEMI patients [10]. In treatment centers without a dedicated catheterization lab or regions with a door-to-balloon time of over 90 minutes, thrombolysis is another effective treatment with comparable length of stay and mortality [10]. After the resolution of the acute thrombosis, management of the different pathologies simultaneously requires careful titration of antithrombotic, antiviral, and anti-inflammatory medications [10].

Since the end of the pandemic, several large-scale clinical trials have found that thromboembolism prophylaxis with apixaban or enoxaparin did not significantly reduce thromboembolic complications or mortality after COVID-19 infection [11-13]. Despite advancements in research and a deeper comprehension of the prothrombotic milieu, current guidelines remain predominantly unchanged due to indistinct evidence. Current guidelines suggest considering prophylactic dose low molecular weight heparin, if not contraindicated, in patients admitted to the hospital or ICU to reduce rates of venous thromboembolism [14,15]. As infection rates decline, it remains imperative to continue to consider COVID-19 infection as a differential diagnosis in patients presenting with recent respiratory infections and associated cardiovascular complications, such as myocardial infarction.

## Conclusions

This case report supports the findings that COVID-19 infection increases thrombosis risk and is related to acute coronary syndrome shortly after infection. Our patient’s STEMI highlights the fact that these patients should be given increased attention when managing their care. COVID-19 has been shown to increase thrombosis risk, and therefore patients with even a limited number of ACS risk factors should be monitored closely in both inpatient and outpatient settings. Prompt management of ACS can decrease mortality. Further studies are needed to determine the long-term outcomes of patients with STEMI and COVID-19.
